# Soil Microbial Biogeography in a Changing World: Recent Advances and Future Perspectives

**DOI:** 10.1128/mSystems.00803-19

**Published:** 2020-04-21

**Authors:** Haiyan Chu, Gui-Feng Gao, Yuying Ma, Kunkun Fan, Manuel Delgado-Baquerizo

**Affiliations:** a State Key Laboratory of Soil and Sustainable Agriculture, Institute of Soil Science, Chinese Academy of Sciences, Nanjing, China; b University of the Chinese Academy of Sciences, Beijing, China; c Departamento de Sistemas Físicos, Químicos y Naturales, Universidad Pablo de Olavide, Seville, Spain; Michigan State University

**Keywords:** future perspectives, recent advances, soil microbial biogeography

## Abstract

Soil microbial communities are fundamental to maintaining key soil processes associated with litter decomposition, nutrient cycling, and plant productivity and are thus integral to human well-being. Recent technological advances have exponentially increased our knowledge concerning the global ecological distributions of microbial communities across space and time and have provided evidence for their contribution to ecosystem functions. However, major knowledge gaps in soil biogeography remain to be addressed over the coming years as technology and research questions continue to evolve. In this minireview, we state recent advances and future directions in the study of soil microbial biogeography and discuss the need for a clearer concept of microbial species, projections of soil microbial distributions toward future global change scenarios, and the importance of embracing culture and isolation approaches to determine microbial functional profiles. This knowledge will be critical to better predict ecosystem functions in a changing world.

## Abstract (precis)

Soil microbial communities are fundamental to maintaining key soil processes associated with litter decomposition, nutrient cycling, and plant productivity and are thus integral to human well-being. Recent technological advances have exponentially increased our knowledge concerning the global ecological distributions of microbial communities across space and time and have provided evidence for their contribution to ecosystem functions. However, major knowledge gaps in soil biogeography remain to be addressed over the coming years as technology and research questions continue to evolve.

## INTRODUCTION

**FIG 1:**
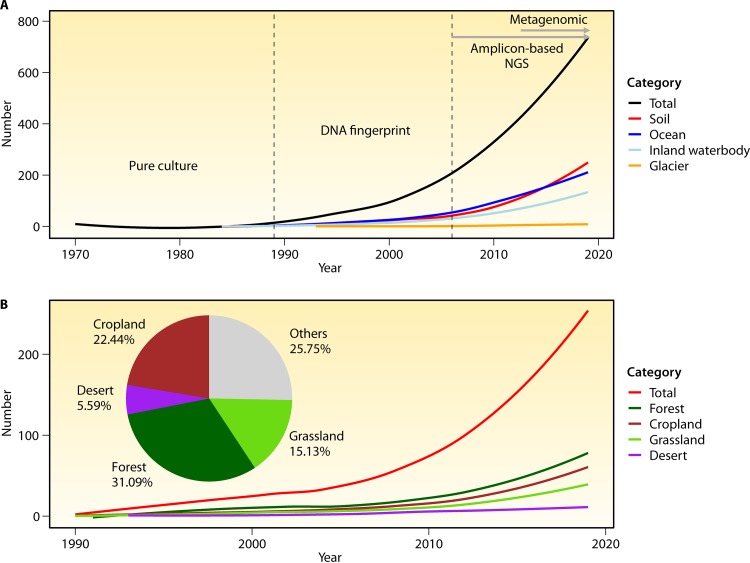
Numbers of published articles in the field of microbial biogeography over time. (A) Numbers of published articles on different ecosystems. Keywords [(microorganism* or microbe* or bacter* or archae* or fung*) and (spatial distribution* or biogeograph* or geographic distance)] were used to search for papers related to microbial biogeography. Additional keywords representing each ecosystem were added, as follows: soil (soil*), ocean (ocean* or sea* or marine*), inland waterbody (river* or lake* or spring* or stream*), and glacier (ice sheet* or glacier*). The vertical dashed lines indicate the critical time points at which the relevant technologies advanced. (B) Numbers of published articles related to all soils and the soils within different ecosystems. Additional keywords representing each ecosystem were added, as follows: forest (forest* or tree* or shrub*), cropland (crop* or farmland* or paddy or agricultur* or rice* or wheat or till* or field soil* or corn* or upland* or peanut), grassland (grassland* or prairie* or turfgrass or grass* or steppe* or meadow* or herb* or sedge*), and desert (desert*). All data were collected from the Web of Science Core Collection. Asterisks represent all possible word endings used for the searches; e.g., fung* represents, fungus, fungi, and fungal.

Soils would not exist without the activity and diversity of millions of soil-resident animals and microorganisms. The aims of soil microbial biogeography are to study the ecological distributions of soil microbial diversity, community composition, and functional traits across space and time from regional to global scales. The study of microbial biogeography is essential to better understand the mechanisms that generate and maintain microbial diversity and that regulate key ecosystem processes, such as nutrient cycling, organic matter decomposition, plant productivity, and public health. Thanks to the development of high-throughput sequencing techniques and bioinformatic analyses (Fig. 1A) (1) and to the growing interest in this topic (Fig. 1B), we are now far from the initial stages of microbial biogeography when Baas-Becking (2) proposed that “everything is everywhere, but, the environment selects” (data in Fig. 1 were collected from the Web of Science Core Collection using the keywords described in the figure legend). Today, we know a lot about the ecological drivers of microbial diversity and community composition across different ecosystem types, including oceans (3, 4), soils (5, 6), and freshwater (7, 8) (Fig. 1A). However, in addition to the critical lack of agreement about the concept of “microbial species,” very little is still known about the hundreds of thousands of microbe-microbe and plant/animal-microbe interactions, which presumably control soil biodiversity and ecosystem functions. Moreover, we know little about the future global distributions of soil microbial taxa under global-change scenarios (especially for the less studied protists), which limits our capacity to predict changes in ecosystem function worldwide. Our capacity to predict changes in microbially driven functions is hampered because of the approaches used. Studies focusing on culturing and whole-genome sequencing are needed to reduce these knowledge gaps. In this minireview, we focus on the biogeography of soil communities and highlight the knowns and unknowns in the field of soil microbial biogeography, from the constantly changing concept of microbial species to future projections of the soil microbiome, and will highlight the advances required to move this field of knowledge forward.

## DISTRIBUTIONS AND ECOLOGICAL DRIVERS OF SOIL MICROBIAL COMMUNITIES AT A GLOBAL SCALE

Studies over the last 2 decades have significantly improved our knowledge of the distributions of soil microbial communities from local, regional, and continental to global scales. From a classic geographical perspective, a negative correlation between distance from the equator and the diversity of plant and animals was largely reported during the last century (9). Similarly, the microbial diversity in marine ecosystems, including bacteria, protists, and planktonic foraminiferans, also exhibited a negative correlation with the global latitudinal gradient (10, 11). However, in soil systems, most studies have not identified the expected trend of soil biodiversity on a global scale. The typical trend of increasing diversity from the poles to the tropics has been partially proven in the Southern Hemisphere. For example, Delgado-Baquerizo et al. (12) found reduced soil bacterial diversity from the equator to Antarctica. However, there is no latitudinal diversity gradient for soil bacteria in the Northern Hemisphere (13, 14). In addition, Bahram et al. (6) found that fungal and bacterial diversity exhibited contrasting patterns across the latitudinal gradient in global topsoils; bacterial, but not fungal, taxonomic diversity was highest in temperate habitats. To date, studies focusing on classic elevation patterns (9) have mostly failed to find a consistent negative or hump-shaped association between elevation and microbial diversity similar to that reported for plants and animals (15–21). Nevertheless, a few studies have reported a declining diversity of soil microbes with increasing elevation, which is similar to the elevation pattern of plant diversity (12, 22). In addition, different microbial taxa may present distinct elevation patterns. For example, Singh et al. (17) observed a single peak pattern for soil bacterial diversity but a double peak pattern for archaeal diversity. However, most latitudinal and altitudinal studies focused on specific mountains ranges and particular locations (e.g., North America or Australia) and focused on a single group of organisms (e.g., bacteria), hampering our capacity to evaluate the changes in microbial diversity across latitudinal and altitudinal gradients comprehensively. Therefore, future global collaborations in soil sampling and data sharing will be particularly important in soil microbial biogeographic studies.

**FIG 2:**
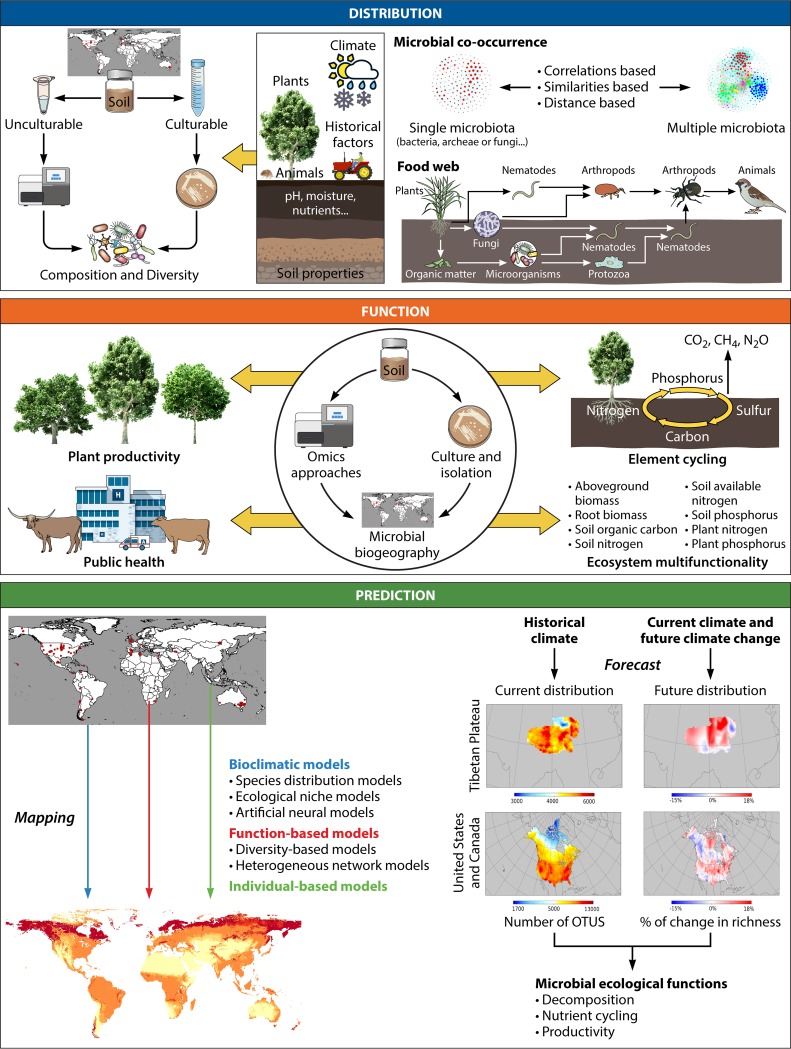
Diagram of the main research fields in soil microbial biogeography that need to be studied spatially and temporally. Boxes with different-colored headings indicate the different research areas. Some diagram elements were modified from the work of Ladau et al. (93). OTUS, operational taxonomic units.

Environmental properties are the most important drivers of the distribution of soil microbial communities globally (Fig. 2). The seminal work by Fierer and Jackson (13) highlighted the importance of soil pH as a fundamental driver of the distribution of bacterial diversity and community composition across contrasting biomes. Delgado-Baquerizo and Eldridge (23) further identified vegetation type and soil carbon content as universal predictors of the diversity of soil bacteria across global biomes. Fierer (24) recently reviewed the major ecological drivers of bacterial diversity. Regarding fungal communities, Tedersoo et al. (25) revealed the role of climate as the major ecological driver and provided the first global study of fungal biogeography. Other less studied global drivers of bacterial and fungal diversity include paleoclimatic legacies (26) and biological warfare (6). Bahram et al. (6) also found that different diversity responses to precipitation and soil pH contributed to the global niche differentiation of soil bacteria and fungi. However, we know much less about the major ecological drivers of other less studied organisms, such as soil protists, mites, nematodes (27, 28), and viruses (29, 30). For example, Bates et al. (31) suggested that climatic conditions likely controlled the distribution of soil protists globally. Liu et al. (32) demonstrated that the distribution of a T4-type phage community in paddy field soils was affected mainly by geographical distance; however, studies focusing on these phages are still rare. In addition, aboveground-belowground interactions and rhizosphere-microbe associations are major drivers of soil microbial diversity on a large spatial scale (Fig. 2). For example, aboveground plant taxonomic and functional attributes help to explain the distribution patterns of microbial biomass, diversity, and community composition (33–37). However, much less is known about the role of belowground plant traits in driving soil microbial distribution. Recently, Ramirez et al. (38) proposed that range-expanding plant species might predict the composition of belowground microbial communities, possibly in association with the belowground traits of these plants (e.g., roots). Animal behavior can also influence soil microbes. For example, ant colonies and mammalian foraging pits can alter the diversity and community composition of soil microbial communities across eastern Australia (39, 40). In addition to the effects of contemporary environmental factors (e.g., climate, soil, plants, and animals), the effects of historical factors (e.g., climatic legacies) (6, 26), and the characteristics of microorganisms themselves (e.g., body size, the ability to colonize, and adhesion) (41) on microbial distribution should be considered (Fig. 2).

Microorganisms are interdependent (42), resulting in some ecologically important but complex interactions, such as antagonistic, competitive, mutualistic, and predator-prey interactions (43) (Fig. 2). This complexity of the interactions among microbial members has been explored increasingly using network analysis (44, 45). The use of correlation networks in microbial ecology (46) have improved our capacity to quantify the level of microbial co-occurrence patterns, understand the drivers of microbial community assembly (e.g., soil carbon and pH and vegetation types) (47–49), and identify highly connected taxa and keystone species (50, 51) across environmental gradients (48). The field of microbial networks is relatively new and should be developed based on the years of experience in studying plant and animal communities (52, 53). However, we still lack strong evidence of the ecological interpretation that exists in network inference, which needs more experimental verification in the future (54).

## LINKING SOIL MICROBIAL BIOGEOGRAPHY TO ECOSYSTEM FUNCTION

Soil biodiversity plays active roles in the regulation of ecological functions and ecosystem services (55–57). A central goal of soil microbial biogeography is to link the distribution of microbial communities with the ecological functions that they support, including single (nutrient cycling, plant productivity, and public health) (58–61) and multiple (ecosystem multifunctionality) functions (56, 57) (Fig. 2). For example, Nelson et al. (61) investigated the global biogeography of microbial N traits (defined as eight N-cycling pathways) and found that some microbial groups seemed to be N-cycling specialists or generalists, suggesting the close relationship between microbial community and N cycling across global soils. In addition, using metagenomic sequencing, Fierer et al. (62) comprehensively surveyed soil microbes and multiple functions in different ecosystems and found that potential soil functional and taxonomic diversity and community composition were highly correlated. Similarly, the richness of bacteria and fungi has been found to drive ecosystem multifunctionality (nutrient cycling, organic matter decomposition, and plant productivity) in regional (63, 64) and global (e.g., dryland) (57) biomes of terrestrial ecosystems. Moreover, microbial community composition was found to regulate the resistance of ecosystem multifunctionality to global change in drylands globally (65). Furthermore, the relationships between soil microbial biodiversity and ecosystem multifunctionality are mediated by climate in the alpine grasslands of the Tibetan Plateau (63). Improving our knowledge of these associations between microbial communities and soil functions is necessary to advance the general prediction for ecological functions at local and global scales. However, further study is needed to better understand how different microbes correlate with soil functions and the underlying mechanisms of how the microbial community drives multiple ecological functions.

Despite the growing amount of data supporting the microbial biodiversity-function relationship, the majority of these studies are based on observational data, and experimental approaches to support the links between microbial taxa and functions are lacking, except in references 56, 64, and 66. For example, experiments have explored the mechanisms of ectomycorrhizal fungi in slowing soil carbon cycling (67), and strictly controlled experiments have been used to directly examine the distribution of trophic links as underlying mechanisms to predict the invasion resistance of plant root-associated bacterial communities against an invading pathogen and the subsequent reduction of disease incidence (68). Recent experimental work suggests that microbial diversity (64) and microbiome complexity (66) determine ecosystem function. However, very little is known about how specific species contribute to ecosystem functioning in the real world. Future experimental work and global initiatives should focus on isolating and culturing soil microbial taxa and on obtaining information via whole-genome sequencing, proteomics, and metabolomics-based approaches to assign specific functions to specific taxa (Fig. 2) (64, 69–72). This information is critical for identifying soil organisms to promote plant production and fight their pests in the field. More importantly, this information will improve our current classification of soil microbes, of which most species remain unclassified. Global initiatives should support taxonomists aiming to dedicate part of their career to culturing and isolating taxa, a fundamental work that is required to move the field of microbial ecology forward but that remains overlooked, partly because it is time-consuming and does not always result in distinguished publications, which hampers researchers’ early careers.

## GLOBAL ATLASES OF SOIL BIODIVERSITY AND THEIR FUNCTIONS UNDER GLOBAL-CHANGE SCENARIOS

A major breakthrough in soil microbial biogeography was the recent appearance of the first global atlases of the abundance or biodiversity of bacteria (23, 73), fungi (74), nematodes (75, 76), earthworms (77), mycorrhizal fungi, and N fixer organisms (78), highlighting the most likely locations containing unknown taxa (79) (Fig. 2). A diverse range of soil characteristics (e.g., soil pH) and climatic conditions has been used to predict and map the global distributions of a myriad of soil organisms at regional (80, 81), national (82, 83), continental (84, 85), and global (25, 73) scales. These efforts have led to the first national atlas of bacterial biodiversity across European Union (EU) member states based on the existing EU-wide soil pH data (84) and the first French national atlas of soil bacterial richness (82). However, more national efforts are needed to map the distributions of soil organisms across their territories, an effort which constitutes the foundation for the national conservation of soil biodiversity. Moreover, global initiatives are needed to further investigate how important land uses, such as agriculture (https://www.globalsustainableagriculture.org), regulate the global distributions of soil organisms.

In addition, to map the current distributions of soil microorganisms, extensive knowledge about the global projections of soil biodiversity under global-change scenarios is missing (Fig. 2). Soil microbial communities are strongly shaped by their surrounding environment; for example, soil microbial diversity and composition are sensitive to drought (86, 87), temperature (88), and fertilization (89). Changes in environmental conditions exert a powerful influence on microbial functions. For example, climate is a predominant driver that regulates litter and root decomposition over large spatial scales (90, 91), mainly via its direct influence on the reaction kinetics of decomposition processes and the decomposer community (92). By leveraging the associations between current bacterial distributions and historical climate data, Ladau et al. (93) predicted that soil bacterial diversity will increase across the majority (about 75%) of the Tibetan Plateau and northern North America if bacterial communities equilibrate with existing climatic conditions. However, we still lack predictions of core microbiomes or keystone species at large spatial scales. In addition, the absolute quantification of microbiota abundance will become more competitive (94), because relative abundance cannot reflect the real microbial composition. In the future, with more detailed background information on microbes, soils, plants, and the climate of the studied sites, we may obtain high-resolution maps of soil microbes. Furthermore, scientists have begun to note the important role of microbial information in improving the resolution of these models (95, 96). The microbial environmental interactome (interactions between microbiomes and their surrounding environment) has shaped the planet’s past and will continue to do so in the future (97). Therefore, the mathematical models used to predict the response and feedback of the ecosystem in the context of global climate change need to incorporate the microbiome data, including the spatial-temporal dynamics of the microbiome and microbial interactions within food webs. Although some efforts have been made to predict the future distributions of soil microbial communities, we still lack the ability to globally predict future soil biodiversity and ecosystem functions into the future.

## FURTHER PERSPECTIVES

Soil microbial biogeography has become a research hot spot in the fields of soil biology and microbial ecology (Fig. 1) (6, 73, 98). Despite the recent advances in molecular techniques and the existence of global efforts, like the Earth Microbiome Project (98, 99), our minireview identified major challenges and research questions in the field of microbial biogeography. Our capacity to address some of these questions remains limited by methodological issues. For example, the differences in data collection and methods from different studies make integrative analysis difficult at regional or global scales. In addition, there are major gaps in current global and temporal sampling data (100) that limit our capacity to predict the distribution of soil microbes spatially and temporally. The present minireview identified some major research areas where studies are needed to move the field of microbial biogeography forward; there is a need for a stronger concept of microbial species, our capacity to generate projections of the soil microbiome toward future global change scenarios needs to be improved, the importance of the complexity within the soil food web should be embraced, and the culture and isolation approaches that determine microbial functional profiles should not be neglected. Such knowledge may help us to cope with the challenges of future environmental changes and improve our ability to accurately predict microbial communities and their function in a changing world. We suggest the following challenges and research opportunities in future microbial biogeographic studies.

### (i) A clear definition of microbial species is still lacking and is essential for biogeographic studies.

Advances in high-throughput sequencing have led to the rapid development of microbial species definitions based on the species’ genealogical, genomic, and phenotypic coherence (101); however, there is not a clear consensus about the concept of “microbial species.” The microbial species definitions based on PCR (phylotype) result in *ad hoc* species groups, which limits our capacity to identify new species and their ecological preferences. In this way, further work based on non-PCR-based methods, high-throughput culturing and identification techniques, and faster microbial isolation and cultivation are needed to enlarge the reference databases (e.g., GenBank, Greengenes, and Silva) and fill the gaps of microbial classification (102). Recently, the genome taxonomy database (GTDB) was developed to provide more pragmatic and objective definitions of taxonomic levels based on sequence distance (103), which is now a primary starting point for gathering sequences to be used in phylogenetic analyses that lead to designations of species and other taxonomic levels.

### (ii) The temporal distribution of microbial communities remains largely unknown.

Information on changes in microbial communities over time (e.g., seasons, years, or much longer time scales) at the large spatial scale is currently a major knowledge gap, although large temporal variances of microbial community compositions have been observed (104). Based on the space-for-time substitution approach (e.g., soil chronosequence, succession, and restoration of ecosystems), it has been found that soil microbial communities may undergo predictable changes over time (5, 105–107). However, such studies are often challenging, because the resolution of DNA-sequencing approaches for temporal dynamics is limited by sequencing both dead and living organisms, and we lack information on microbial communities in globally distributed locations and across multiple years. Thus, for example, considering the existence of relic DNA in samples as a confounding factor is key in detecting fine-scale temporal patterns in microbial communities using DNA approaches (108, 109). Moreover, future global efforts, including existing global cooperative efforts like CLIMIFUN, NEON, LUCA, and NUTNET, should aim to monitor temporal variations in soil microorganisms and to set up additional sites in poorly studied regions from polar, tropical, and arid regions and in continents like Africa, Antarctica, and South America. Such accumulated samples and data over years will help us to better understand the change in microbial communities under future global-change scenarios.

### (iii) Organisms should be engineered to support human development.

Future advances in microbial biogeography associated with the use of synthetic biology, new approaches for microbial culturing, and multiple bio-omics (e.g., metatranscriptomics, metaproteomics, and metabolomics) may help us to harness the soil microbiome to promote crop production and health in a changing world. We are still far from knowing what functions are being conducted by every single microbial species and their contribution to terrestrial functions, yet synthetic biology approaches and engineering of microorganisms have been postulated to boost ecosystem restoration, rhizosphere-driven crop yield, and pest control (110, 111), to fight global environmental change (112, 113), and even to aid the terraformation of other moons and planets to make them more similar to Earth (114, 115).

### (iv) Researchers should make predictions for the soil microbiome into the future.

Improving the prediction accuracy of models is critical for microbial mapping. First, soil samples must be collected from more types of habitats and as many locations as possible to enrich the database. Second, soil microbial communities are temporally dynamic; therefore, it is necessary to understand microbial variations at different time scales. Third, not only the microbial diversity and community composition but also other information, such as microbial interactions (microbe-microbe, microbe-plant, microbe-host interactions), need to be considered and integrated into the model to improve our capacity to predict changes in key ecosystem functions (e.g., carbon storage) on a global scale. These accuracy-improved models may be further used to predict the temporal-spatial dynamics in soil biodiversity and ecosystem functions under changing environments, which will aid the conservation of soil biodiversity and the display of ecological functions under future climate change.
